# Defining the roles of the N-terminal region and the helicase activity of RECQ4A in DNA repair and homologous recombination in *Arabidopsis*

**DOI:** 10.1093/nar/gkt1004

**Published:** 2013-10-29

**Authors:** Susan Schröpfer, Daniela Kobbe, Frank Hartung, Alexander Knoll, Holger Puchta

**Affiliations:** ^1^Botanical Institute II, Karlsruhe Institute of Technology, Hertzstrasse 16, Karlsruhe 76187, Germany and ^2^Institute for Biosafety in Plant Biotechnology, Julius Kühn Institute (JKI), Erwin-Baur-Strasse 27, Quedlinburg 06484, Germany

## Abstract

RecQ helicases are critical for the maintenance of genomic stability. The *Arabidopsis* RecQ helicase RECQ4A is the functional counterpart of human BLM, which is mutated in the genetic disorder Bloom’s syndrome. RECQ4A performs critical roles in regulation of homologous recombination (HR) and DNA repair. Loss of RECQ4A leads to elevated HR frequencies and hypersensitivity to genotoxic agents. Through complementation studies, we were now able to demonstrate that the N-terminal region and the helicase activity of RECQ4A are both essential for the cellular response to replicative stress induced by methyl methanesulfonate and cisplatin. In contrast, loss of helicase activity or deletion of the N-terminus only partially complemented the mutant hyper-recombination phenotype. Furthermore, the helicase-deficient protein lacking its N-terminus did not complement the hyper-recombination phenotype at all. Therefore, RECQ4A seems to possess at least two different and independent sub-functions involved in the suppression of HR. By *in vitro* analysis, we showed that the helicase core was able to regress an artificial replication fork. Swapping of the terminal regions of RECQ4A with the closely related but functionally distinct helicase RECQ4B indicated that in contrast to the C-terminus, the N-terminus of RECQ4A was required for its specific functions in DNA repair and recombination.

## INTRODUCTION

RecQ helicases are conserved in all organisms and have multiple functions in DNA recombination, repair and replication. RecQ helicases contribute to the maintenance of genomic stability by regulating recombination between homologous DNA molecules and processing DNA intermediates. Mutations in three human RecQ helicase genes (*BLM*, *WRN* and *RECQL4*) cause the severe hereditary diseases Bloom’s syndrome (BS), Werner’s syndrome and Rothmund–Thomson syndrome, which are associated with a predisposition to cancer, premature aging and developmental defects, respectively (1–3).

Since the discovery of *recQ* in *Escherichia coli* (4), multiple RecQ helicases have been identified in many different classes of organisms [see reviews (5,6)]. A maximum number of seven RecQ genes have been identified in the plant species *Arabidopsis thaliana* and *Oryza sativa* (7–9). Among the RecQ helicases identified in *Arabidopsis*, RECQ4A and RECQ4B exhibit the highest similarity to the eukaryotic RecQ homologues BLM in humans and Sgs1 in *Saccharomyces cerevisiae* (7). There is a strong conservation of the typical RecQ domain structure among the BLM, Sgs1 [see review (6)], RECQ4A and RECQ4B helicases. These conserved structural domains consist of a helicase domain, a RecQ C-terminal (RQC) domain and a helicase and RNase D C-terminal (HRDC) domain. The gene pair *RECQ4A* and *RECQ4B* arose from a recent segmental duplication on chromosome 1 within the *Brassicaceae* family (7,10). Interestingly, despite a high degree of sequence identity between RECQ4A and RECQ4B, a functional divergence occurred and only RECQ4A seems to share similar functions with Sgs1 and BLM. The loss of Sgs1, BLM and RECQ4A leads to comparable phenotypes with respect to DNA recombination and repair. *BLM*-defective cells (BS cells), *sgs1* mutants and *recq4A* mutants are hypersensitive to DNA-damaging agents such as methyl methanesulfonate (MMS) (10–12). BS cells from BS patients exhibit a characteristic phenotype in which an elevated frequency of sister chromatid exchanges (SCEs) is observed (13). Like BS cells, *sgs1* and *recq4A* mutants also exhibit a hyper-recombination phenotype, which suggests a role for these proteins in homologous recombination (HR) regulation (10,14,15). Recently, the biological role of a RECQ4A/RECQ4B homologue RECQL4 was characterized in rice (16). Similar to RECQ4A in *Arabidopsis*, the loss of RECQL4 in rice resulted in hypersensitivity to DNA-damaging agents and exhibited a hyper-recombination phenotype (16). Mutants of *Arabidopsis RECQ4B* are not sensitive to DNA-damaging agents and do not display a hyper-recombination phenotype; in contrast, a reduction of the frequency in HR in somatic cells was reported (10). Furthermore, in contrast to RECQ4B, RECQ4A seems to be involved in the maintenance of telomeres during meiosis and is associated with meiotic recombination intermediates leading to a reduction in fertility (17).

In mitotic cells, HR is required for the efficient repair of double-stranded DNA breaks (DSBs) and single-stranded DNA gaps, which can arise during S-phase in the presence of DNA damage and blockage of replication fork progression [see review (18)]. BLM and Sgs1 are required for multiple steps in HR and act as both pro- and anti-recombinogenic factors. A conserved function of RecQ helicases in HR is the dissolution of recombination intermediates like double Holliday Junctions (dHJs) mediated by the RTR complex. In the first dissolution step, the branch migration activity of a RecQ helicase is required to generate a hemi-catenane intermediate, which is then resolved by a type IA topoisomerase, resulting exclusively in a non-crossover product [see review (19)]. It was previously shown that human BLM interacts with this complex through topoisomerase 3α (TOP3A) and two OB-fold containing structural proteins RMI1 and RMI2 (20–23). In *S. cerevisiae*, the RTR complex consists of Sgs1, Top3 and Rmi1 (24–27). RECQ4A was shown to be involved in the RTR complex in *Arabidopsis* together with TOP3A and RMI1 by analysis of genetic interactions (10,28).

A direct protein interaction between Sgs1 and Top3 or BLM and TOP3A, respectively, was demonstrated *in vivo* and *in vitro*. In yeast, the N-terminus of Sgs1 mediates the interaction with Top3 (29–32). Similarly, the interaction of BLM with TOP3A is mapped to an N-terminal domain, which is essential for the correct localization of TOP3A and for the suppression of SCEs (33). Another TOP3A binding domain was identified in the C-terminus of the BLM helicase (34). Both the N- and C-terminal TOP3A binding domains are only weakly conserved at the sequence level, but they are able to interact with Top3 of yeast *in vivo*.

Previous studies have shown that at least some functions of Sgs1 and BLM are due to their ability to process DNA substrates mediated by their helicase activity. The loss of Sgs1 helicase activity results in hypersensitivity to DNA damage caused by MMS and hydroxyurea (HU) (12,32,35). Furthermore, it has been shown that the helicase activity of Sgs1 and BLM is required for the suppression of HR (12,32,35–37).

Here, we have used a complementation approach to define the roles of the N-terminal region and the helicase activity of RECQ4A with regard to its functions in DNA repair and recombination *in vivo*. By *in vitro* analysis we could demonstrate helicase activity and fork regression ability within the RECQ4A core domain. Moreover, by swapping the N- and the C-terminal domains of RECQ4A with the respective parts of RECQ4B, we were able to demonstrate that only the N- but not the C-terminus is required for RECQ4A-specific functions.

## MATERIALS AND METHODS

### *In vitro* analysis of RECQ4A

The coding sequence for amino acids 420–983 of RECQ4A (NM_100968.2), which contains the helicase and the RQC domains, was cloned in a modified pET-Duet-1 vector (Novagen) giving rise to an expression construct with the following additional (bold) N- and C-terminal sequences: **MMH**TYTEGS …FPSSVKV**HVGTHHHHHHSTSAWSHPQFEK**. Thus, a His-tag and a StrepII-Tag were added to the C-terminus. In a second expression construct (HD), a point mutation was introduced in the Walker A box leading to GGG**M**SLT instead of GGG**K**SLT, as already characterized for HsWRN, AtRECQ2 and AtRECQ3 (38–40). This corresponds to K481M of RECQ4A. For expression *E. coli* strain ER2566 RIL was used. This is an expression strain created by selecting transformands of ER2566 (NEB) with the plasmids extracted from BL21-CodonPlus(DE3)-RIPL (Stratagene) for the uptake of the pSC101 based RIL plasmid with streptomycin/spectinomycin resistance. ER2566 RIL was transformed with either the 420–983 or the HD expression construct. For each construct LB (5 g/l tryptone, 2.5 g/l NaCl, 2.5 g/l yeast extract, 200 µl/l 10 M NaOH) supplemented with 100 µg/µl ampicillin was inoculated (1 colony/100 ml) and incubated O/N at 28°C with 60 rpm. Afterwards, incubation was at 200 rpms. After reaching an OD of 0.6 at 600 nm, temperature was lowered to 16°C, and expression was induced with 0.2 mM IPTG for 3 h. The cells were harvested by centrifugation and frozen. The purification was at 4°C except for the washing step at room temperature. Cells were lysed by lysozyme and sonication in BW200 (100 mM Tris, 200 mM NaCl, 0.1% Tween20, 10 mM thioglycerol, pH 8), and the supernatant after centrifugation was applied to Strep-Tactin Superflow (IBA) gravity flow columns. Before elution with desthiobiotin, washing was successively with BW200 and RT buffer (100 mM Tris, 500 mM NaCl, 10 mM MgCl_2_, 5 mM ATP, 10 mM thioglycerol, pH 8 used at room temperature). The elution fractions were applied to a Ni^2+^-loaded HiTrap Chelating HP column (GE Healthcare) equilibrated in buffer A (BW200 + 50 mM imidazole). For elution, the column was developed with a linear gradient of imidazole up to 400 mM, and the fractions were mixed with the same volume of glycerol. RECQ4A protein-containing samples were pooled, aliquoted and stored at −80°C. Activity was analysed in assay buffer at 37°C [40 mM Tris acetate (pH 8), 50 mM KAc, 6 mM DTT, 1.8 mM ATP, 5.4 mM MgCl_2_ and 50 µg/ml BSA]. ATPase activity was determined photospectrometrically as decrease of OD340 in assay buffer with 25 µM nts calf thymus DNA, 3 mM phosphoenolpyruvate, 20 U/ml lactate dehydrogenase, 20 U/ml pyruvate kinase and 0.25 mM NADH. Approximately 5 nM enzyme and respective dilutions were used. Helicase activity was analysed after 20 min with 150 pM 3′ overhang generated by annealing 32-P-labelled 5′ ATTAA GCTCT AAGCC ATGAA TTCAA ATGAC CTCTT ATCAA 3′ and 5′ TTGAT AAGAG GTCAT TTGAA TTCAT GGCTT AGAGC TTAAT TTTTT TTTTT TTTTT T 3′ or 100 pM of a synthetic replication fork essentially as described (41). Approximately 13 nM enzyme and respective dilutions were used. As replication fork allowing a fork regression event, HomF 30/30 was prepared as described (42).

### Plant lines and plant growth conditions

The mutant lines *recq4A-4* [GABI_203C07, (10)], *top3A-1* and *top3A-2* [SALK_139357, GABI_476A12 (28)] have been previously described. For reproduction of the plant lines, plants were grown in soil (1:1 mixture of Floraton 3 and Vermiculit) under long day conditions (16 h light/8 h dark) at 22°C. For sterile plant culture, seeds were surface sterilized with 70% ethanol and 4% sodium hypochlorite solution and rinsed in ddH_2_O. After stratification overnight at 4°C, sterilized seeds were plated on selective or pure germination medium (GM: 4.9 g/l Murashige & Skoog-medium, 10 g/l saccharose, pH 5.7, 7.6 g/l plant-agar) and incubated in a plant growth chamber (Percival Scientific, CU-36L4; 16 h light at 22°C/8 h dark at 20°C).

### Cloning of RECQ4A constructs for plant transformation

The binary plasmid pPZP221 (43) was used as the basis of construction for plant transformation vectors containing different *RECQ4A* constructs. The basic composition of the *RECQ4A* constructs included a promoter region, a coding region (cDNA) and a terminator region. The promoter/UTR (798 bp upstream of the start codon of *RECQ4A*) and terminator (406 bp downstream of the stop codon of *RECQ4A*) sequences were amplified from genomic DNA. To amplify the coding regions, cDNA was used for the PCR template. The construct *RECQ4A-HD*, which contains a point mutation leading to an amino acid substitution of lysine 481 to methionine (GGG**M**SLT instead of GGG**K**SLT), was created by site-directed mutagenesis PCR followed by an overlap extension PCR. To create homologous regions required for assembly of the single fragments by In-Fusion cloning or overlap extension PCR, the purified PCR products were extended with the appropriate overhang primer. Detailed information about the cloning PCRs, primer combinations, primer sequences and PCR templates is given in Supplementary Table S1.

pPZP221-*RECQ4A,* -*RECQ4A-ΔN*, -*RECQ4A-ΔN-HD*, -*RECQ-(4B)4A* and -*RECQ-4A(4B)* were generated by In-Fusion technology using the In-Fusion Advantage PCR Cloning Kit (Clontech). In a single In-Fusion reaction, the PCR products of the respective promoter, the coding sequence and the terminator were assembled in order and simultaneously integrated into pPZP221 linearized with BamHI, which was directed by the accessory and appropriate sequence homologies.

The *RECQ4A-HD* construct was assembled by successive overlap extension PCRs and first integrated into pCR-BluntII-TOPO (Invitrogen), followed by subcloning into pPZP221 using BamHI and PstI restriction sites.

The integrity of all pPZP221 derivatives was verified by sequencing (GATC Biotech AG).

### Generation of transgenic plant lines

The pPZP221 derivatives were stably transformed into *Arabidopsis* plant lines by the *Agrobacterium*-mediated floral dip method (44) using the GV3101::pMP90 *Agrobacterium* strain. Transgenic plants containing the transformed T-DNA were identified by plating on solid GM selection medium (60 mg/l gentamycin) in T1. In plant generation T2, statistical analyses [critical value χ^2^ (1;0.95)] of the segregation behaviour of the plant lines were performed to identify single-locus lines. Plants containing the respective homozygous T-DNA were selected in generation T3 on selection medium. To verify the genotype of the transformed plant lines in T4, the transgenic lines were plated on different GM selection media (for *recq4A-4*: 10 mg/l sulfadiazine, for IC9 recombination reporter: 10 mg/l hygromycin).

### Determination of the recombination frequency

To determine inter-molecular recombination events, plants homozygous for the HR reporter construct IC9 were used as previously described (10,45). Briefly, 40 7-day-old seedlings from an axenic plant culture were transferred into halved petri dishes containing 10 ml liquid GM. After eight additional days in liquid culture, the seedlings were histologically stained (10). Subsequently, the blue sectors on each plantlet were quantified using a binocular microscope. This HR assay was repeated independently at least three times. The mean values of the respective plant lines were normalized to the level of the mutant line.

### Sensitivity assays

Sensitivity to genotoxic agents was assessed as previously described (10). Ten 7-day-old seedlings from an axenic plant culture were transferred into pure liquid GM (5 ml GM for assays without genotoxic agent; 4 ml GM for assays with genotoxic agent exposure). One day later, the plants were treated with 1 ml of the appropriate genotoxic solution to achieve the respective final concentration of the genotoxic agent (MMS: 60 ppm, 80 ppm; cisplatin: 5 µM, 10 µM). After additional 13 days of incubation in the growth chamber, the plants were taken out and dried with a paper towel. Subsequently, the fresh weight of these plants was determined. For each line, the fresh weight of the respective treated plants was normalized to that of the untreated plants.

## RESULTS

### Lysine 481 is essential for the ATPase and helicase activity of RECQ4A

To define functional regions of the protein RECQ4A *in planta*, phenotypical analysis of *recq4A-4* mutant lines transformed with different *RECQ4A* cDNA constructs were performed. In an initial set of experiments, the role of the RECQ4A helicase function in DNA repair and DNA recombination was addressed. In general, it is possible to obtain mutant proteins devoid of helicase function by the introduction of specific point mutations. However, no data were available for generating a helicase-dead RECQ4A ORF. Therefore, a biochemical approach was chosen to obtain the required information. By sequence alignments a highly conserved lysine was identified at position 481; it was located in the Walker A motif of the helicase domain of RECQ4A that is essential for the ATPase activity of other helicases (46). It was previously reported that substitution of this lysine with methionine in the human RecQ helicase WRN and the RECQ2 and RECQ3 helicases from *Arabidopsis* leads to a loss of ATPase activity and a loss of helicase activity (38–40).

**Figure 1.**
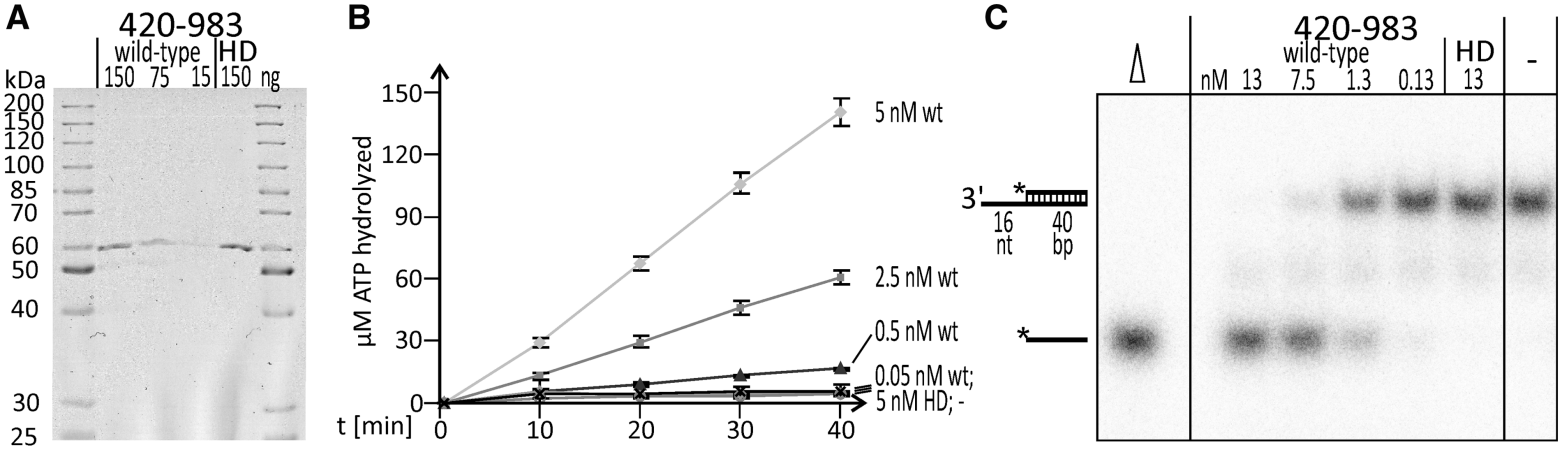
Demonstration that the catalytic core of RECQ4A, but not the corresponding K481M variant (HD), has ATPase and helicase activity. **(A)** Colloidal Coomassie stained 10% SDS–PAGE showing different dilutions of purified RECQ4A-420-983 (wild type) and RECQ4A-K481M-420-983 (HD). The loaded quantities are given in ng. **(B)** ATPase activity of different dilutions of purified RECQ4A-420-983 (wild type, wt) and RECQ4A-K481M-420-983 (HD). The final concentrations of enzymes in the reaction are indicated. − marks a reaction without enzyme (negative control) **(C)** Autoradiogram showing strand displacement (helicase) activity of different dilutions of purified RECQ4A-420-983 (wild type) and RECQ4A-K481M-420-983 (HD). The final concentrations of enzymes in the reaction are indicated. − marks a reaction without enzyme (negative control). Δ shows a heat-denatured sample.

The catalytic core of RECQ4A spanning the helicase and the RQC domains and the corresponding Walker A variant (HD) were expressed in *E. coli* and purified via C-terminal affinity tags. Analysis by SDS–PAGE and Western Blotting revealed a single band with an apparent molecular weight of ∼60 kDa (Figure 1A). The difference between the actual and calculated sizes of the protein (67 kDa) is most likely due to electrophoretic migration abnormalities. Electrophoretic migration abnormalities seem to occur quite often with DNA processing proteins and were also observed, e.g. with MUS81/EME1A,1B (47). The concentrations of the 420–983 fragment and the HD fragment were adjusted and controlled by dilution. An ATPase assay clearly demonstrated the ATPase activity of the catalytic core of RECQ4A and the abolishment of this activity by the amino acid substitution from lysine to methionine in the Walker A Motif (HD, K481M) (Figure 1B). Furthermore, helicase activity of the catalytic core of RECQ4A could also be demonstrated via a strand displacement assay (Figure 1C). The helicase activity was strong because unwinding of the 3′ overhang substrate could be detected, even in the 1:100 dilution samples (0,13 nM). As expected from the ATPase data, the HD version was similar to the negative control in that it exhibited no detectable helicase activity. Thus, the K481M amino acid substitution completely abolishes both ATPase and helicase activity.

### Generation of plant lines for *in vivo* experiments

**Figure 2.**
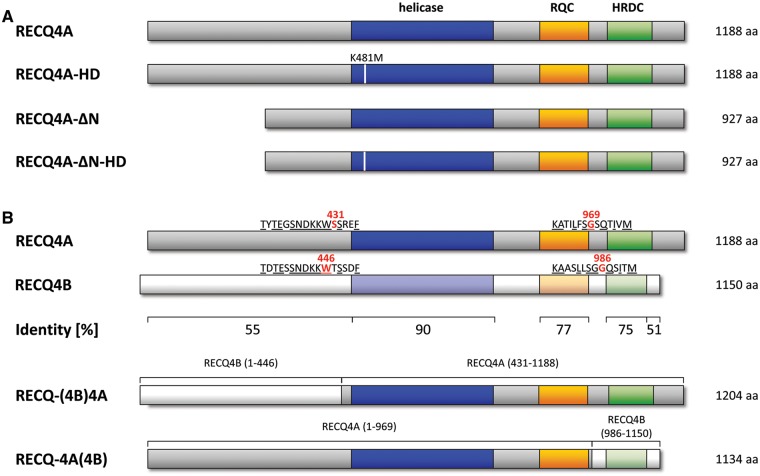
Schematic representation of the recombinant RECQ4A variants. **(A)** The respective *RECQ4A* constructs were transformed into plant lines. RECQ4A-HD contains an amino acid substitution (K481M, lysine to methionine at position 481) in the Walker A motif of the helicase domain. In RECQ4A-ΔN, the N-terminal amino acids 2–262 of RECQ4A are deleted. RECQ4A-ΔN-HD is truncated in the N-terminus and contains the amino acid substitution in the Walker A motif. **(B)** The level of conservation of RECQ4A and RECQ4B is indicated by the percentage of identical amino acids in the respective protein region. The chimeric protein RECQ-(4B)4A contains amino acids 1–446 from RECQ4B at the N-terminus adjacent to amino acids 431–1188 of RECQ4A. RECQ-4A(4B) contains N-terminal amino acids 1–969 of RECQ4A and 986–1150 of RECQ4B, including the HRDC domain. Protein sequences of positions of the exchange between RECQ4A and RECQ4B in the chimeric variants are depicted.

For complementation experiments with the *recq4A-4* mutant, different *RECQ4A* constructs were cloned including a full-length wild-type construct (*RECQ4A*) and different variants of the *RECQ4A* ORF in which individual domains were modified or deleted [*RECQ4A-HD, RECQ4A-ΔN*, *RECQ4A-ΔN-HD*, *RECQ-(4B)4A* and *RECQ-4A(4B)*], respectively. To mimic the natural expression pattern and level of RECQ4A variants (Figure 2) in plants, the 798-bp-long genomic sequence located upstream of the start codon of *RECQ4A* was used as a promoter in the *RECQ4A* constructs. This promoter region includes the complete 5′-UTR, as well as the first intron of *RECQ4A*, located in the 5′-UTR. 5′-UTR introns were identified as regulators of protein expression in *Arabidopsis* (48). The 406-bp-long genomic region downstream of the stop codon of *RECQ4A* was selected as terminator. By homology-directed cloning methods, the different DNA fragments were fused to each other and integrated into the binary plasmid pPZP221. The resulting T-DNA was stably transformed into the *Arabidopsis recq4A-4* mutant and the wild-type line by *Agrobacterium*-mediated transformation, both of which harbour the IC9 recombination reporter in its homozygous state. Complementation analyses were performed with at least five genetically different plant lines that all contained the transgenic *RECQ4A* construct at a single homozygous locus.

### Complementation of the hypersensitive *recq4A* mutant phenotype

**Figure 3.**
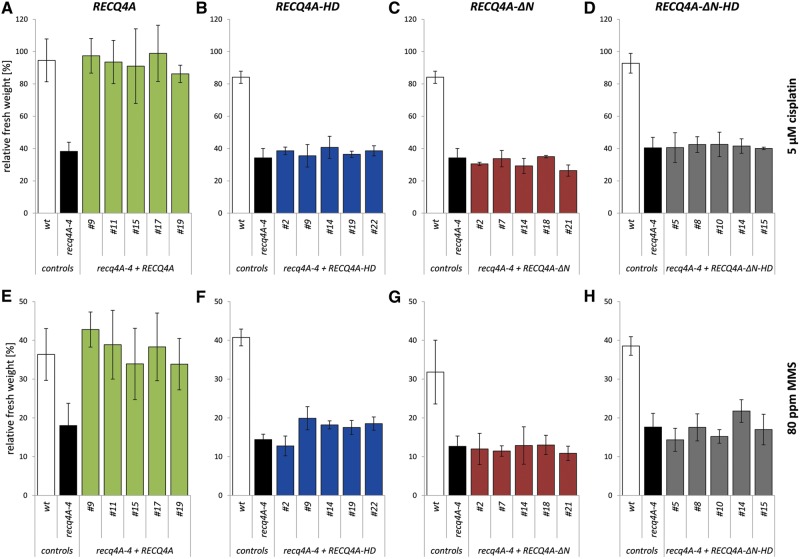
The role of the N-terminus and helicase activity of RECQ4A in response to cisplatin and MMS-induced DNA damage. The fresh weight of 10 seedlings after 13 days of genotoxin treatment [5 µM cisplatin **(A–D)**, 80 ppm MMS **(E–H)**] was determined. The relative fresh weight of each line is given as percentage and was calculated from the relation of fresh weight of each line at a respective genotoxin concentration to the fresh weight of the same line without genotoxin treatment. Each assay was performed at least three times as described, and the mean values including standard deviations (error bar) are depicted. The expression of the wild-type construct *RECQ4A* in *recq4A-4* mutant background (*recq4A-4 + RECQ4A*, green) enables a full complementation of the elevated sensitivity of *recq4A-4* against cisplatin (A) and MMS (E). The constructs *RECQ4A-HD* (blue, B, F), *RECQ4A-ΔN* (red, C, G) and *RECQ4A-ΔN-HD* (grey, D, H) cannot complement the hypersensitivity of *recq4A-4* to cisplatin and MMS.

The *recq4A-4* mutant is hypersensitive to treatment with cisplatin (10), which mainly induces intra-strand DNA cross-links (CLs) (49). Furthermore, the loss of RECQ4A also leads to a hypersensitivity to the DNA methylating agent MMS (10). To analyse the sensitivity of plant lines carrying the recombinant *RECQ4A* constructs, a liquid medium assay was used and the fresh weight of 3-week-old plantlets treated with two different concentrations of the respective genotoxic agent was determined. After treatment with 5 µM cisplatin, the mutant *recq4A-4* exhibited a strong reduction in fresh weight to 38% in comparison to the wild-type line (95% fresh weight) (Figure 3A). *recq4A-4* mutant lines transformed with the *RECQ4A* wild-type construct (Figure 2A) exhibited a relative fresh weight comparable to the wild-type level after treatment with 5 µM cisplatin (Figure 3A). A full complementation of the hypersensitivity to cisplatin was also observed for a second concentration of 10 µM cisplatin (Supplementary Figure S1A). Furthermore, the elevated sensitivity of *recq4A-4* to 60 and 80 ppm MMS could be rescued in *recq4A-4* mutant lines containing the *RECQ4A* wild-type construct, as shown in Figure 3E (80 ppm) and Supplementary Figure S1E (60 ppm). The *RECQ4A* wild-type construct fully complemented the hypersensitive phenotype of the *recq4A-4* mutant. Thus, the promoter used for the complementation approach guaranteed the expression of recombinant RECQ4A protein in a way in which all functions of the endogenous RECQ4A wild-type protein in repair of cisplatin and MMS-induced DNA damage *in planta* could be fulfilled.

As a control experiment, the relative fresh weight of three wild-type lines transformed with the full length *RECQ4A* construct was determined and was not different from the wild-type weight after treatment with cisplatin or MMS (data not shown). These results indicate that the transformation process or the transgene itself did not diminish the capacity of these plants to repair DNA damage.

### The helicase activity of RECQ4A is important for its role in DNA repair

After defining K481 in the Walker A box as an essential amino acid for the helicase activity, the effects of the physical presence of a helicase-defective recombinant protein RECQ4A-HD (Figure 2A) as defined by our biochemical analysis were analysed *in vivo*. In relation to the wild-type line, the relative fresh weights of *recq4A-4* and *recq4A-4* mutant lines transformed with the *RECQ4A-HD* construct were reduced to a similar extent after treatment with cisplatin [Figure 3B (5 µM); Supplementary Figure S1B (10 µM)] or MMS [Figure 3F (80 ppm); Supplementary Figure S1F (60 ppm)]. Thus, the hypersensitivity of *recq4A-4* could not be complemented by the expression of the helicase-defective *RECQ4A-HD* variant. A negative complementation on the sensitivity of plants was excluded in additional experiments: wild-type plants transformed with the *RECQ4A-HD* construct behaved like wild-type plants in sensitivity assays performed with cisplatin and MMS (data not shown). In summary, these data demonstrate that the helicase activity of RECQ4A is important for its function in the repair of intra-strand CLs and methylated DNA.

### Deletion of the RECQ4A N-terminus does not affect the viability of plants but results in a DNA repair defect

The RECQ4A homologues Sgs1 and BLM in yeast and mammals, respectively, directly interact with the type IA topoisomerase 3/3α in the RTR complex, which promotes the dissolution reaction of HJs to suppress the formation of crossovers. For both homologues, the N-terminal portion of the respective RecQ helicase mediated this direct protein–protein interaction (29,30,33,34). Despite weaker sequence conservation of the N-termini of these RecQ homologues in yeast, mammals and plants, a functional conservation in relation to the interaction with Top3/TOP3A protein is likely. For functional studies of the N-terminal region of the plant homologue RECQ4A, the effects of deleting the N-terminal amino acids 2–262 were analysed (RECQ4A-ΔN, Figure 2A). A deletion of the N-terminal Top3 interaction domain of Sgs1 leads to a phenotype comparable to that of a *top3* mutants in yeast (35). In this study, viable plant lines transformed with the *RECQ4A-ΔN* construct were established and their growth did not differ from that of wild-type lines. The *top3A* mutant phenotypes in plants [lethality or an obvious growth defect (28)] were not mimicked in *recq4A-4* mutant lines expressing *RECQ4A-ΔN* (Supplementary Figure S2).

To further analyse the role of the RECQ4A N-terminus, the effect of the deletion of the N-terminus on the sensitivity of plants to DNA-damaging agents was determined. In comparison to wild type, *recq4A-4* mutant lines transformed with *RECQ4A-ΔN* exhibited an elevated sensitivity to cisplatin [Figure 3C (5 µM); Supplementary Figure S1C (10 µM)] and MMS [Figure 3G (80 ppm); Supplementary Figure S1G (60 ppm)] which is comparable to the sensitivity of the *recq4A-4* mutant. Thus, the hypersensitivity of *recq4A-4* could not be restored by the expression of the *RECQ4A-ΔN* variant. This observation points to an essential function of the N-terminal portion of RECQ4A in response to DNA damage induced by cisplatin or MMS, similar to the results obtained for RECQ4A helicase activity. Consistent with these findings, *recq4A-4* plant lines expressing *RECQ4A-ΔN-HD*, a RECQ4A variant lacking the N-terminus and defective in helicase activity (Figure 2A), also showed a hypersensitivity like the mutant and plant lines only missing one of the regions after treatment with cisplatin [Figure 3D (5 µM); Supplementary Figure S1D (10 µM)] or MMS [Figure 3H (80 ppm); Supplementary Figure S1H (60 ppm)].

### Both the N-terminal region and the helicase activity contribute to the suppression of HR

Like *sgs1* mutants and *BLM*-defective cells, the loss of the plant homologue RECQ4A also leads to a strong increase in the frequency of recombination events (10,14). For complementation analysis, the recombination reporter IC9 (45,50) was used, which contains two non-functional parts of the β-glucuronidase gene (*GUS*) with overlapping homologous regions in such an orientation that restoration of the reporter gene is possible only by recombination using the sister chromatid or the homologous chromosome. The frequency of recombination events, which were visible as blue sectors on the plantlets upon histological staining, was quantified.

**Figure 4.**
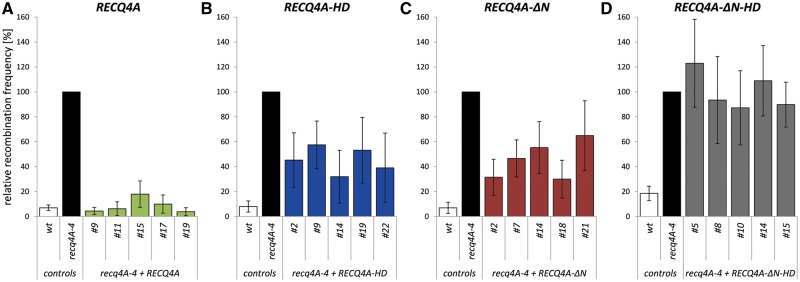
The role of the N-terminus and helicase activity of RECQ4A in suppression of hyper-recombination. The frequency of HR events was determined using the recombination reporter line IC9 and depicted as the relative recombination frequency normalized to the level of *recq4A-4*. Each assay was performed at least three times, and the mean values including standard deviations (error bar) are depicted. The expression of the wild-type construct *RECQ4A* in *recq4A-4* mutant background (*recq4A-4 + RECQ4A*, green) fully complements the hyper-recombination phenotype of *recq4A-4* **(A)**. Mutant lines transformed with *RECQ4A-HD* (blue, **B**) and *RECQ4A-ΔN* (red, **C**) exhibit an intermediate recombination frequency, which implies a partial complementation of the elevated recombination frequency. The construct *RECQ4A-ΔN-HD* (grey, **D**) cannot complement the hyper-recombination phenotype of *recq4A-4*.

In comparison to the wild-type line, the relative recombination frequency of the mutant *recq4A-4* was elevated by 14-fold, whereas *recq4A-4* mutant lines transformed with the *RECQ4A* wild-type construct exhibited a reduced recombination frequency which is comparable to that of the wild type (Figure 4A). In a control experiment it was shown that the additional expression of RECQ4A in the wild-type line mediated by the transformed *RECQ4A* construct did not affect the recombination level in comparison to wild type (data not shown). Thus, the reduction of recombination events by the construct is specific for the mutant line. In summary, the full complementation of the hyper-recombination mutant phenotype shows that the recombinant protein RECQ4A can completely substitute for the endogenous RECQ4A protein function involved in the suppression of HR events between chromatids.

Because the helicase activity and the N-terminal portion of RECQ4A are both crucial for the response to intra-strand CLs and DNA damage induced by methylation, it was interesting to analyse whether the same holds true for the regulation of recombination. Several independently transformed lines expressing the *RECQ4A-HD* variant in a *recq4A-4* mutant background displayed an intermediate recombination frequency that was 2- to 3-fold reduced in comparison with the *recq4A-4 *line but was 4- to 7-fold enhanced in comparison with the wild type (Figure 4B). Apparently, the helicase-defective protein RECQ4A-HD can perform some functions in suppression of HR, leading to a partial complementation of the hyper-recombination *recq4A-4* phenotype. Furthermore, these results also suggest that the helicase activity of RECQ4A is required to a certain extent for suppression of HR.

Notably, the *recq4A-4* mutant lines transformed with the *RECQ4A-ΔN* construct, missing the N-terminal part, also exhibited an intermediate recombination phenotype (Figure 4C).

The previous results indicated that both the N-terminal region and the helicase activity play a role in the suppression of somatic HR. However, both could be involved in the same type of reaction that would require those features of RECQ4A for some but not all ways of HR suppression as detected by our assay system. On the other hand, both features might contribute to the suppression phenotype in an independent manner. In this case, the loss of both regions would lead to a complete loss of suppression. To discriminate between these possibilities, a construct missing both the N-terminal region and the helicase activity of RECQ4A was generated (Figure 2A). *recq4A-4* mutant plant lines transformed with *RECQ4A-ΔN-HD* showed a recombination frequency that was increased in comparison with wild type as well as the mutant expressing the *RECQ4A-ΔN* or *RECQ4A-HD* construct, respectively. However, the recombination frequency of *recq4A-4* plants expressing *RECQ4A-ΔN-HD* was indistinguishable from that of the mutant (Figure 4D).

Thus, these analyses demonstrate that the helicase activity and the N-terminus of RECQ4A are both involved in the suppression of HR. Moreover, these functional features seem to be either independent or only partially overlapping, suggesting a multifunctional role for RECQ4A in HR regulation.

### The RECQ4A core domain is able to catalyse fork regression

**Figure 5.**
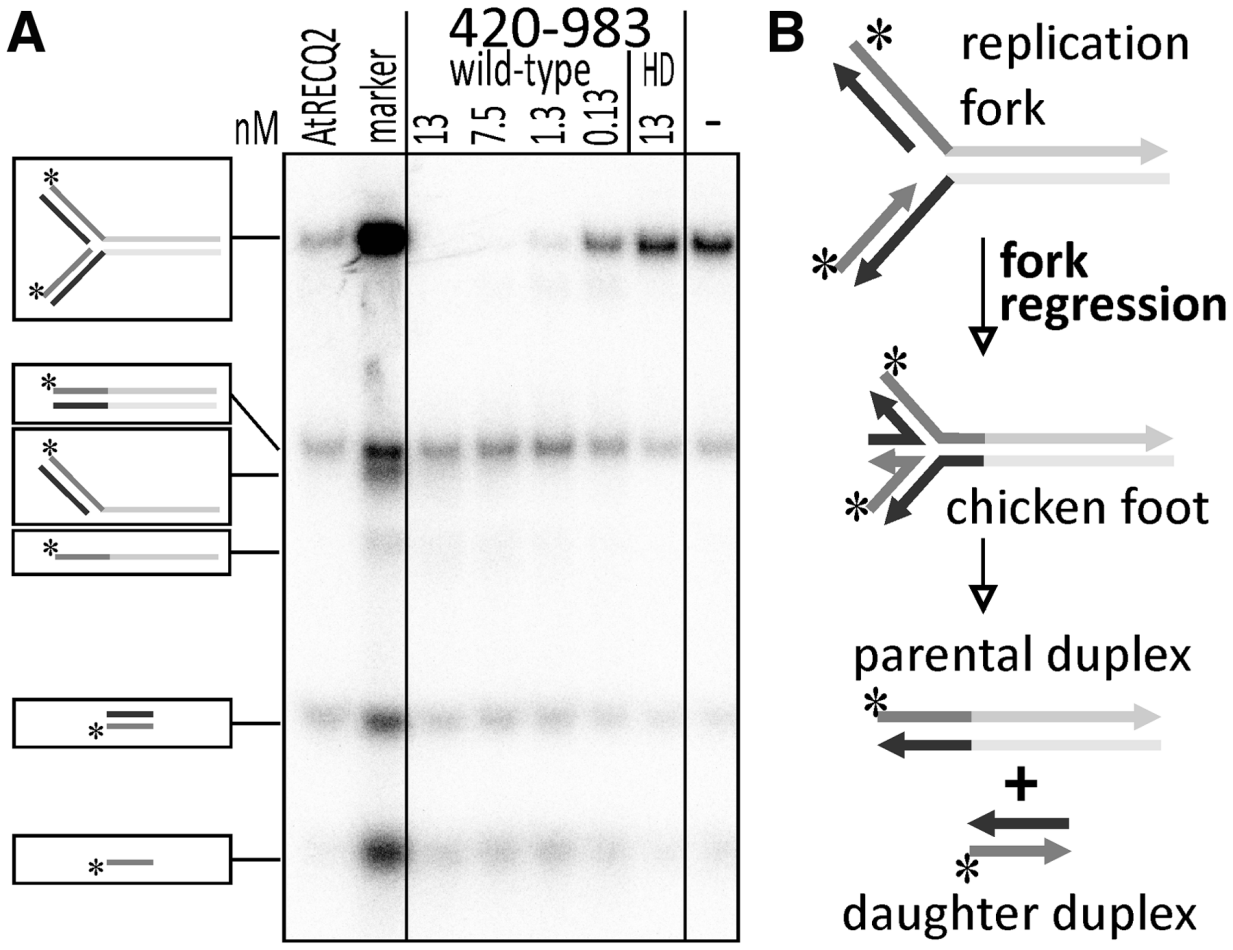
Fork regression by the catalytic core of RECQ4A. **(A)** Autoradiogram of the reaction of different dilutions of purified RECQ4A-420-983 (wild type) and RECQ4A-K481M-420-983 (HD) with the HomF 30/30 synthetic replication fork separated by 12% TBE-PAGE. The final concentrations of enzymes in the reaction are indicated in nM. As a control, purified AtRECQ2 was used. − marks a reaction without enzyme (negative control). **(B)** Schematic illustration of the chicken foot intermediate formed by fork regression, which is further processed to parental and daughter duplexes and can be detected in the autoradiogram.

Some somatic HR events may arise due to stalled replication forks. One way to resolve stalled replication forks caused by blockage of leading strand synthesis is replication fork regression followed by template switching. Because both the helicase activity and the N-terminus of RECQ4A were involved in suppression of HR, it was interesting to test whether the helicase activity itself was sufficient to promote fork regression. Therefore, the reaction of the catalytic core fragment of RECQ4A with a replication fork that allows fork regression (42) was analysed (Figure 5). Because of previous experiments showing that RECQ2 from *Arabidopsis* can catalyse this reaction on a similar substrate (40), RECQ2 was included as a control. As expected, RECQ2 produces parental and daughter duplexes, which are the products indicative of fork regression. Interestingly, the catalytic core of RECQ4A lacking the N-terminus and the HRDC domain can also process the replication fork. The main products are parental duplexes, daughter duplexes and single-stranded daughter strands. The HD version is not able to process the substrate, as the observed pattern is exactly the same as the pattern found in the control without enzyme. Thus, the helicase activity but not the N-terminus of RECQ4A is required for replication fork regression.

### Swapping of the N- and C-termini of RECQ4A with RECQ4B indicates that only the N-terminus defines the functional specificity

The genes *RECQ4A* and *RECQ4B* of *Arabidopsis* arose from a recent segmental duplication on chromosome 1 inside the *Brassicaceae* family. In spite of high sequence identity between both proteins, mutants exhibit different phenotypes with respect to the genetic interactions of double mutants of *top3A* and *mus81* (10,51). In contrast to RECQ4A, the RECQ4B helicase is not involved in the repair of cisplatin and MMS-induced DNA damage or in the suppression of HR. The level of conservation varies in different parts of RECQ4A and RECQ4B (Figure 2B). The characteristic RecQ domains display a high degree of identical amino acids between both RecQ helicases: 90% in the helicase domain, 77% in the RQC domain and 75% in the HRDC domain. Outside of the conserved domains, a lower sequence identity of 55% in the N-terminus and 51% in the C-terminus could be observed.

**Figure 6.**
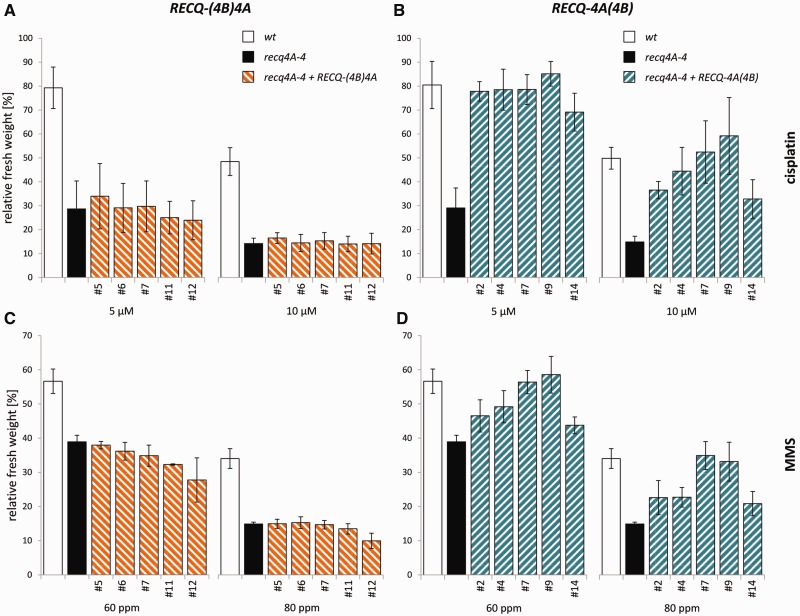
Complementation analysis of the hypersensitivity of *recq4A-4* lines transformed with chimeric RECQ4A/4B constructs. The fresh weight of 10 seedlings after 13 days of genotoxin treatment [cisplatin **(A, B)**, MMS **(C, D)**] was determined. The relative fresh weight of each line is given as percentage and was calculated from the relation of fresh weight of each line at a respective genotoxin concentration to the fresh weight of the same line without genotoxin treatment. Each assay was performed at least three times as described, and the mean values including standard deviations (error bar) are depicted. The expression of the chimeric construct *RECQ-(4B)4A* containing *RECQ4B* sequences in the N-terminus in *recq4A-4* mutant background [*recq4A-4 + RECQ-(4B)4A,* orange] cannot complement the elevated sensitivity of *recq4A-4* to cisplatin (A) and MMS (C). Mutant lines containing the chimeric constructs *RECQ-4A(4B)* with *RECQ4B* sequences in the C-terminus (cyan, B, D) show a full or a partial complementation of the hypersensitivity to cisplatin and MMS, respectively, which is dependent on the respective line and the concentration of the genotoxin.

**Figure 7.**
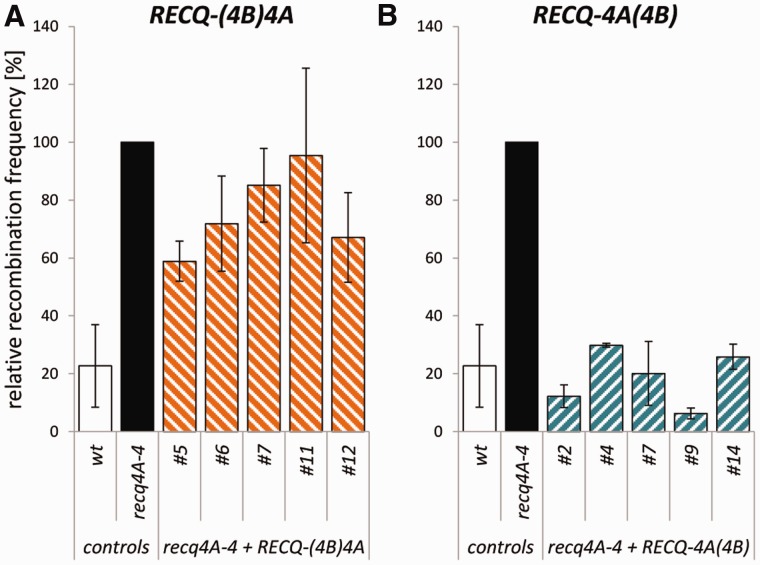
Complementation analysis of the elevated HR frequency of *recq4A-4* with chimeric RECQ4A/4B proteins. The analysed plant lines contain the recombination reporter IC9, which allows the determination of the frequency of inter-molecular HR events, which are depicted as the relative recombination frequency normalized to the level of *recq4A-4*. Each assay was performed at least three times, and the mean values including standard deviations (error bar) are depicted. Mutant lines expressing the chimeric *RECQ-(4B)4A* construct [*recq4A-4* + RECQ-(4B)4A, orange] show a partial or no complementation of the hyper-recombination phenotype, which is dependent on the respective line **(A)**. The expression of *RECQ-4A(4B)* in mutant background (cyan) fully complements the elevated recombination rate of *recq4A-4* **(B)**.

To test if the more divergent sequences in the N- and C-termini are responsible for the functional differentiation of both RecQ helicases, the complementation ability of chimeric RECQ4A variants containing either the N- or the C-terminus of RECQ4B (Figure 2B) were analysed. The positions for the exchange of RECQ4A with RECQ4B sequences were placed in parts of the protein exhibiting high amino acid identities. The chimeric RECQ-(4B)4A contains amino acids 1–446 from RECQ4B at the N-terminus adjacent to amino acids 431–1188 of RECQ4A. In *recq4A-4* mutant lines transformed with the construct *RECQ-(4B)4A*, an elevated sensitivity to cisplatin (Figure 6A) and MMS (Figure 6C) could be observed which is comparable to the sensitivity of *recq4A-4*. Therefore, there was no complementation of the hypersensitive *recq4A-4* phenotype by RECQ-(4B)4A. For somatic HR, dependent on the respective *recq4A-4* line containing the *RECQ-(4B)4A* construct, in some lines a partial complementation or no complementation of the elevated recombination frequency could be observed, as represented by an intermediate or a recombination frequency indistinguishable from *recq4A-4* mutant plants, respectively (Figure 7A). This result is in agreement with previous analysis (see above) in which partial complementation of the hyper-recombination phenotype could be achieved to a similar extent for the RECQ4A-ΔN variant. Thus, the partial complementation by RECQ-(4B)4A seems to rely on the RECQ4A helicase function of the chimera. In summary, our results based on a complementation analysis with RECQ-(4B)4A indicate that the N-terminus of the duplicated sister RECQ4B cannot fulfil functions of the RECQ4A N-terminus in DNA repair and HR suppression.

The effects of an exchange of the C-terminus of RECQ4A were examined by the chimeric construct RECQ-4A(4B), which contains N-terminal amino acids 1–969 of RECQ4A and 986–1150 of RECQ4B, including the HRDC domain (Figure 2B). In sensitivity assays performed with 5 µM cisplatin, the hypersensitive mutant phenotype could be rescued in all of the five tested *recq4A-4* lines containing *RECQ-4A(4B)* (Figure 6B). By testing a higher concentration of 10 µM cisplatin (Figure 6B) and MMS (Figure 6D, 60 and 80 ppm), two of the analysed lines (#7, #9) still displayed a full complementation, whereas three of these lines showed a partial complementation of the hypersensitive phenotype. This finding may be due to a lower protein level of RECQ-4A(4B) in lines #2, #4 and #14 when compared to the other two lines and correlating with the total amount of DNA damage. However, a full complementation of the elevated sensitivity against cisplatin and MMS could be shown in two *recq4A-4* lines containing the chimeric construct *RECQ-4A(4B)*. Furthermore, the hyper-recombination phenotype of *recq4A-4* could be fully suppressed to wild-type levels by the transformation of *RECQ-4A(4B)* in all tested plant lines (Figure 7B). Thus, in contrast to RECQ-(4B)4A, the chimeric protein RECQ-4A(4B) can fulfil all functions of the endogenous RECQ4A during the repair of cisplatin and MMS-induced DNA damage and in HR regulation.

Our analysis demonstrates that the functional differentiation of both RecQ helicases RECQ4A and RECQ4B is not due to the sequence divergence within the C-terminus, including the HRDC domain. In contrast, the N-terminal sequence of RECQ4A defines its functional specificity in comparison to RECQ4B.

## DISCUSSION

### Functional analysis of RECQ4A in *Arabidopsis*

Mutations in the human RecQ helicase gene BLM lead to BS, a hereditary disease associated with genomic instability and a predisposition for cancer (1). Functional analysis of BLM in living mammals is difficult because homozygous mutations of *BLM* cause embryonic lethality in different mouse models (52,53). In contrast to mammalian cell culture and the model system yeast, *in vivo* studies of the BLM orthologue RECQ4A could be performed in a complex, living multicellular organism using the model plant *Arabidopsis*.

We have confirmed our previous results regarding the hypersensitive and hyper-recombination *recq4A-4* phenotype by complementation using a stably transformed, randomly integrated T-DNA construct containing *RECQ4A* cDNA between the natural promoter and terminator. Our studies were performed with different plant lines to minimize misinterpretations resulting from the T-DNA integration process, as an intragenic T-DNA insertion locus can affect other relevant genes. Furthermore, the position effect and T-DNA copy numbers could have an influence on the expression level of the transgene (54,55). To exclude secondary effects, such as negative complementation by the expression of recombinant proteins, each T-DNA was also transformed into wild-type lines. Because we were able to demonstrate full complementation of the *recq4A-4* phenotypes by the transformed *RECQ4A* wild-type construct, we concluded that the respective promoter construct guarantees an appropriate pattern and level of expression. Thus, the pre-requisite for analysis of RECQ4A variants was given.

### RECQ4A and its interaction with the RTR complex

The dissolution of recombination intermediates like dHJs is a critical step in HR and seems to be a conserved functional interaction between RecQ helicases and topoisomerase 3/3α from bacteria to humans [see review (56)]. Mutants of the RTR complex partner Top3 in yeast exhibit a slow growth phenotype, which can be suppressed by deletion of Sgs1 in *S. cerevisiae* or Rqh1 in *Schizosaccharomyces pombe* (25,57,58). Comparable to these observations in yeast, the lethal phenotype of the *Arabidopsis* mutant *top3A* can be rescued by an additional mutation in *RECQ4A*, indicating that the lethality of *top3A* is dependent upon the activity of RECQ4A (10). To explain the dramatic phenotype of the *top3/top3A* single mutant, it was postulated that the RecQ helicase irreversibly generates a toxic DNA intermediate, which remains unresolved in the absence of the topoisomerase (25).

For Sgs1 in yeast and BLM in humans, an N-terminal Top3/TOP3A interaction domain is conserved (25,29,30,33,34). The deletion of the N-terminal Top3 interaction domain of Sgs1 leads to a severe phenotype comparable to the *top3* phenotype, and this phenotype is dependent upon the helicase function of Sgs1 (35). In contrast to yeast, we have shown that the deletion of the N-terminus of RECQ4A in plants did not lead to *top3A* mutant lethality or growth defects. This finding cannot be due to no functional RECQ4A-ΔN being produced, because we could show that the truncated RECQ4A-ΔN variant can fulfil some roles with regard to the suppression of somatic HR. Moreover, the RECQ4A protein with the RECQ4B N-terminus also showed partial complementation of the suppression of HR, but no growth defect was observed. In spite of the absence of the *top3* phenotype, the function of RECQ4A is strongly disturbed by the deletion of the N-terminus, which is represented by hypersensitivity and an elevated recombination frequency when compared to wild type. We assume that the N-terminus of RECQ4A is important for the RTR complex to process recombination intermediates.

One possible explanation for the differences between plant and yeast might be that other regions of RECQ4A may also be involved in this TOP3A interaction. In the human BLM protein, but not in the yeast Sgs1 protein, a further TOP3A interaction domain could be mapped to the C-terminus (34). Another possibility is that the deleted N-terminal region of RECQ4A might not be exclusively responsible for the interaction with TOP3A, but also for the interaction with other partners. In yeast, the interaction with Rmi1 was also mapped to the N-terminus of Sgs1 (59). Human BLM not only interacts with TOP3A and RMI1 but also with RMI2 (21,23,60), a further partner of the mammalian RTR complex. The N-terminus of RECQ4A might also mediate a direct protein interaction with the plant homologues of RMI1 and RMI2. Recently we were able to demonstrate by functional domain analysis of the *Arabidopsis* RMI1 protein, that mutations/deletions of parts of the protein that are equivalent to the parts required for the interaction of BLM with the RMI1 homologue in mammals results in a defect in repair and recombination similar to the phenotype of the *recq4A* mutant. This can be taken as indication that indeed the interaction of RMI1 and RECQ4A is required for processing of DNA repair intermediates in plants, too (61).

### RECQ4A and perturbed replication forks

Helicase function as well as the N-terminus of RECQ4A are essential for its functions in the repair of DNA damage resulting from alkylation of DNA by the genotoxic agent MMS. This effect could also be shown for Sgs1 via complementation analysis in yeast (12,32,35). As shown in this study, the helicase activity and the N-terminus of RECQ4A are also crucial for the response to intra-strand CLs caused by treatment with cisplatin. Although the nature of these types of DNA damage is primarily different, both might induce stalling of the replication fork during DNA replication in S-phase. This notion is in accordance with current models linking RecQ functions to replication fork progression. For example, cells from BS patients display a delay in S-phase and accumulate abnormal replication intermediates (62–64). Furthermore, BLM levels are regulated in a cell-cycle-dependent manner with enrichment during S-phase (65,66). It was also reported that BLM and Sgs1 accumulate at sites of stalled replication forks (67,68), and that this localization is dependent upon a functional RTR complex (21). In *sgs1* and *top3* mutants, the mechanisms required to restart replication after HU treatment are defective (69). In yeast, the loss of Sgs1 leads to an accumulation of unprocessed X-shaped DNA structures at blocked replication forks during S-phase (70,71). These structures seem to be HR-mediated HJ containing DNA intermediates derived from a stalled replication fork, which accumulate in RTR-defective cells (6,19,71,72). Because of the described sensitivity pattern and the comparisons to yeast and mammalian homologues, a role of the plant RECQ4A at perturbed replication forks is most likely.

### RECQ4A regulates HR by different mechanisms

For the repair or tolerance of DNA damage at stalled replication forks, a number of different pathways were proposed [see review (73)]. Some of these pathways are based on HR mechanisms such as template-switching via strand-invasion into the undamaged sister chromatid or damage repair by generation of a one-sided DSB. However, other template-switching pathways require fork regression. It is possible that the action of RECQ4A is involved in these pathways in response to replicative stress.

The expression of a helicase-defective RECQ4A leads to a partial complementation of the elevated recombination frequency in *recq4A-4* lines. RECQ4A therefore seems to play different roles in HR suppression, some of which are helicase-dependent and some of which are helicase-independent. It is possible that RECQ4A is involved in fork regression via its helicase activity. *In vitro* studies could show that human BLM can catalyse the regression of replication forks by generating HJ-containing chicken-foot structures, and furthermore, BLM can also promote the reversal of regressed replication forks (74–76). Consistently, BLM was shown to be involved in the efficient restarting mechanism of replication, which requires its helicase activity (77). The loss of RECQ4A does not lead to hypersensitivity to Mitomycin C (MMC), another DNA-crosslinking agent that mainly causes inter-strand CLs (10,78), which cannot be repaired or tolerated by fork regression. These *in vivo* observations fit with our conclusion that RECQ4A plays a role in fork regression. Our hypothesis is strongly corroborated by *in vitro* demonstration that the core domain of RECQ4A lacking the N-terminus is able to regress an artificial replication fork (see Figure 5).

Interestingly, there are conflicting reports on the role of helicase activity of RECQ homologues in suppressing recombination in eukaryotes. Early reports indicated that no complementation of the elevated recombination frequencies could be achieved in yeast *sgs1* mutants and BS cells by a helicase-defective protein (Sgs1-hd or BLM-HD, respectively), suggesting an essential function of the helicase activity of Sgs1 and BLM in recombination suppression (12,32,36,37). However, more recently, Weinstein and Rothstein (35) reported that an intermediate recombination suppression phenotype existed using a different yeast strain that contains a helicase-defective *Sgs1* allele at the endogenous *Sgs1* locus. This is in line with our results of a partial complementation of the hyperrecombination phenotype with a helicase-defective RECQ4A variant in plants. Hence, a conservation of the helicase-dependent and -independent function in HR suppression between plants and yeast can be assumed. Lo *et al.* (79) already postulated that helicase-dependent functions of Sgs1 were involved in fork regression and branch migration of dHJs in the dissolution reaction. Helicase-independent functions of Sgs1 were explained by stabilization of the RTR complex, whereupon the formation of the hemi-catenane can be processed by a passive migration of the dHJ caused by physical forces (79). Cejka *et al.* (24) also reported a structural role for Sgs1 during the dissolution reaction, which is independent of the helicase activity of Sgs1.

As discussed earlier, the deletion of the N-terminus of RECQ4A in the complementation construct only leads to a partial reversion of the elevated recombination frequency of the mutant. Furthermore, RECQ4A variants missing both the helicase activity and the N-terminus cannot complement the hyper-recombination phenotype of the mutant. This observation indicates that the helicase activity and the N-terminus are involved in different reactions to suppress somatic HR events. In line with this hypothesis, reports have suggested that only a partial suppression of the elevated SCE frequency of BS cells could be observed in complementation experiments with a BLM variant deleted in the N-terminus in a mammalian system (33,37).

In summary, our data indicate that RECQ4A plays an important role in the regulation of recombination at perturbed replication forks and is involved in different DNA damage tolerance pathways. Although RECQ4A could possibly promote fork regression in a manner dependent upon its helicase activity, it is also an integral component of the RTR complex that possesses important functions in the processing of recombination intermediates arising during HR.

### The N-terminus is important for defining the specificity of RECQ4A

A common characteristic of plants is that gene pairs or even gene families can be found in their genomes that originated from complete or partial genome duplications or hybridizations that occur regularly during evolution. These genes will have a redundant function initially, but in the long run, often sub- or neo-functionalization takes place, which may also help to stabilize the presence of several paralogues in the genome (80). This development will result in a situation in which the duplicated genes can no longer complement each other’s functions. Looking at the enzyme machinery of *Arabidopsis* involved in DNA repair and HR, one can find examples of the different developmental states of gene pairs: in the case of *BRCA2A* and *BRCA2B* (81), both genes still have redundant functions in DNA repair and HR, whereas in the case of *BRCC36A* and *BRCC36B* (82), overlapping functions occur in DNA repair but not in HR. *RECQ4A* and *RECQ4B* clearly differ in their function in DNA repair as well as in HR (10,51). One is tempted to speculate that specific sub- or even a neo-functionalization of *RECQ4B* seems to have occurred during evolution. Based on previous results regarding sensitivity to DNA-damaging agents, recombination behaviour and genetic interactions with *top3A* and *mus81* in the mutant, it seems likely that *RECQ4A* retained at least most of the functions of a putative *RECQ4* ancestor. In this report, we addressed the question of which regions of the protein are crucial for the specific functions of RECQ4A via complementation experiments with chimeric or mutated proteins. By exchanging the N-terminus of RECQ4A with the corresponding N-terminal region of RECQ4B, we found that the N-terminus of RECQ4B cannot mediate the functions of the RECQ4A N-terminus in HR suppression and DNA repair. In contrast, a chimeric RECQ4A variant containing the C-terminal region including the conserved HRDC domain of RECQ4B could fully complement the elevated recombination frequency and the hypersensitivity to MMS and cisplatin of the *recq4A-4* mutant.

Our results indicate that the functional difference of both RecQ helicases is mainly defined by differentiation of the N-terminus, a further sign of the important role of the N-terminus of RECQ4A. Presumably, RECQ4B is not able to interact with components of the RTR complex due to the sequence differences in its N-terminus. It is most likely that the characteristic properties of the N-terminus of a putative RECQ4 ancestor were conserved during evolution in RECQ4A but that these properties were lost in the RECQ4B gene.

## SUPPLEMENTARY DATA

Supplementary Data are available at NAR Online.

## FUNDING

German Research Foundation DFG [Pu 137/10, Pu 137/11]; European Research Council ERC [ERC-2010-AdG_20100317 COMREC]; The Young Investigator Group [YIG 9-109 to D.K.] received financial support by the, ‘Concept for the Future’ of Karlsruhe Institute of Technology (KIT) within the framework of the German Excellence Initiative. Funding for open access charge: European Research Council.

*Conflict of interest statement.* None declared.

## Supplementary Material

Supplementary Data
