# Supplementary material for: Defining the roles of the N-terminal region and the helicase activity of RECQ4A in DNA repair and homologous recombination in Arabidopsis
Source: Nucleic Acids Res. 2013 Oct 29;42(3):1684–97. doi: 10.1093/nar/gkt1004 (PMC3919593; doi:10.1093/nar/gkt1004)
Supplement: Supplementary Data [file supp_42_3_1684__index.html]

Defining the roles of the N-terminal region and the helicase activity of RECQ4A in DNA repair and homologous recombination in Arabidopsis — Defining the roles of the N-terminal region and the helicase activity of RECQ4A in DNA repair and homologous recombination in Arabidopsis — Supplementary Data 

# Defining the roles of the N-terminal region and the helicase activity of RECQ4A in DNA repair and homologous recombination in *Arabidopsis*

## Supplementary Data

files

**Files in this Data Supplement:**

- Supplementary Data - pdf file
