# Supplementary material for: Defining the roles of the N-terminal region and the helicase activity of RECQ4A in DNA repair and homologous recombination in Arabidopsis
Source: Nucleic Acids Res. 2013 Oct 29;42(3):1684–97. doi: 10.1093/nar/gkt1004 (PMC3919593; doi:10.1093/nar/gkt1004)
Supplement: Supplementary Data [file supp_gkt1004_nar-01849-m-2013-File008.pdf]

SUPPLEMENTARY MATERIAL

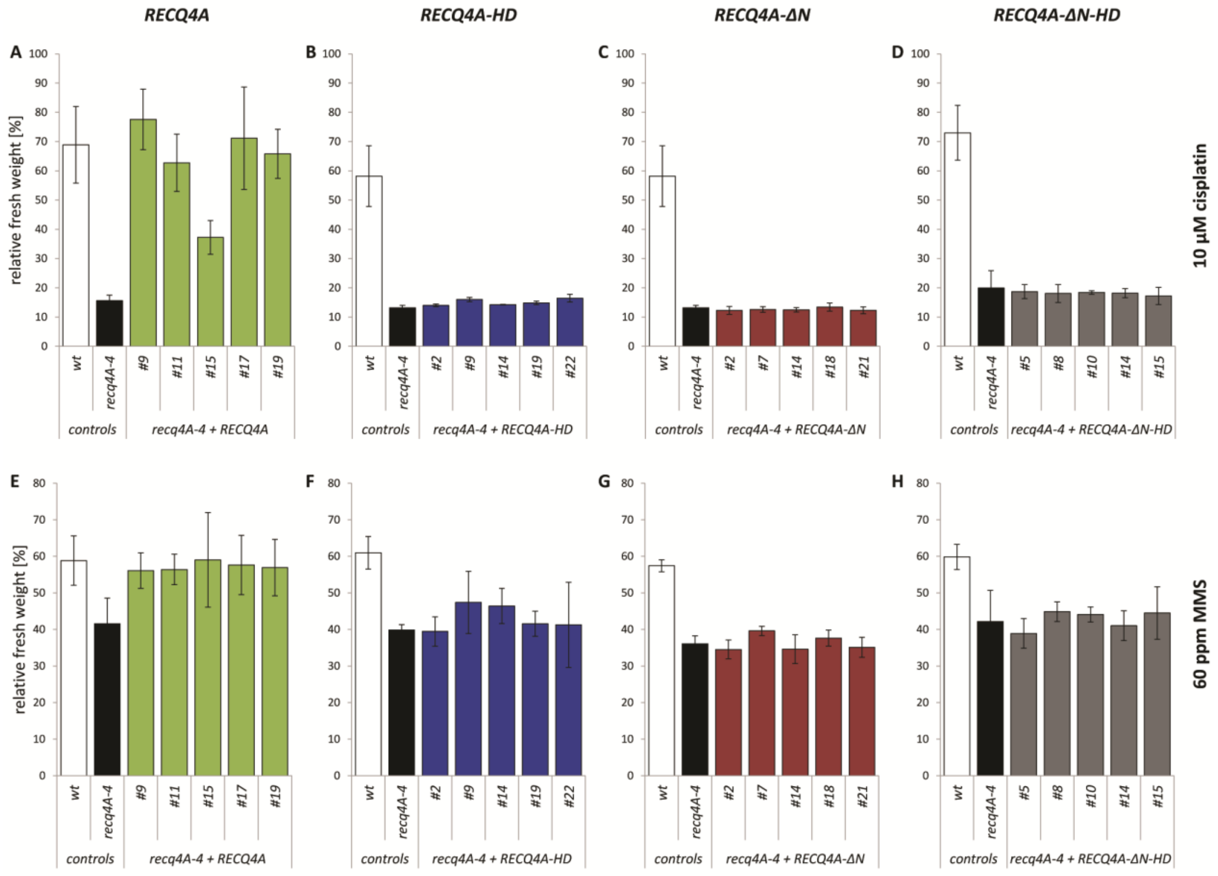

Figure S1. Functional analysis of the N-terminus and helicase activity of RECQ4A in the repair of cisplatin (10 µM) and MMS (60 ppm)-induced DNA damage. The fresh weight of 10 seedlings after 13 days of genotoxin treatment (10 µM cisplatin (A-D), 60 ppm MMS (E-H)) was determined. The relative fresh weight of each line is given as percentage and was calculated from the relation of fresh weight of each line at a respective genotoxin concentration to the fresh weight of the same line without genotoxin treatment. Each assay was performed at least three times as described and the mean values including standard deviations (error bar) are depicted. The expression of the wild type construct *RECQ4A* in *recq4A-4* mutant background (*recq4A-4* + *RECQ4A*, green) enables a full complementation of the elevated sensitivity of *recq4A-4* to cisplatin (A) and MMS (E). The constructs *RECQ4A-HD* (blue, B, F), *RECQ4A-ΔN* (red, C, G) and *RECQ4A-ΔN-HD* (grey, D, H) cannot complement the hypersensitivity of *recq4A-4* to cisplatin and MMS.

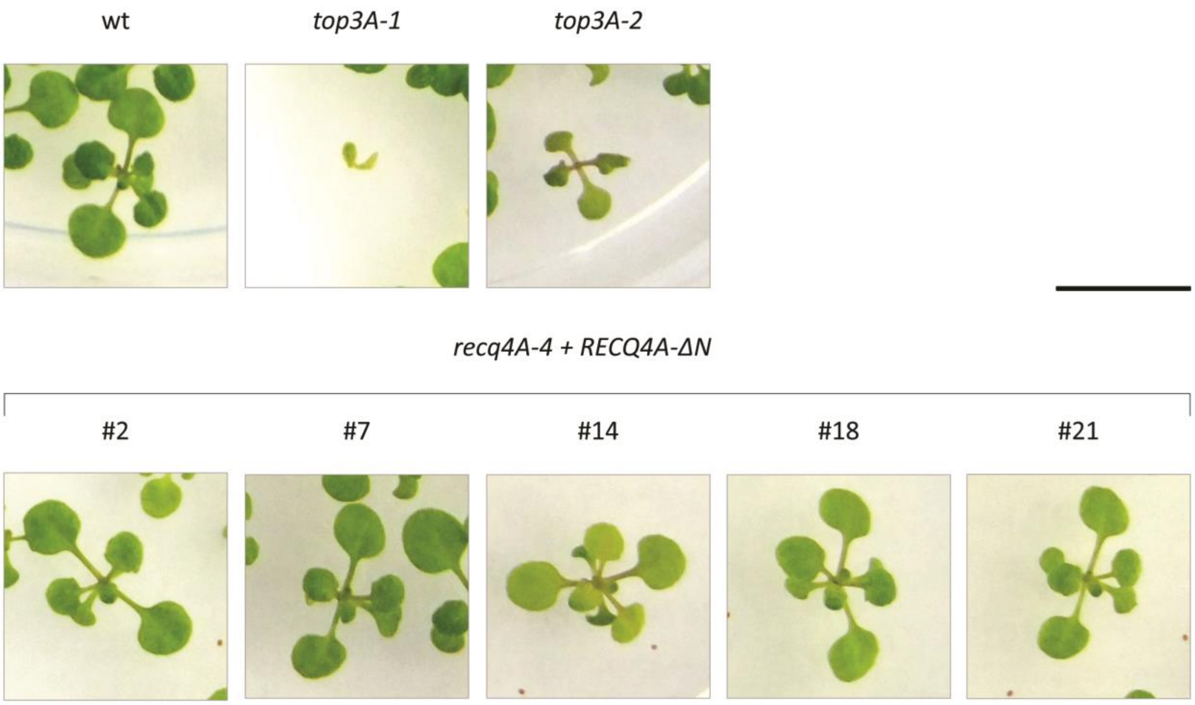

Figure S2. Growth phenotypes of *top3A* mutant lines and *recq4A-4* lines transformed with *RECQ4A-ΔN.* Images of representative 16-days-old plant seedlings of wild type, homozygous *top3A-1* and *top3A-2* mutants as well as different *recq4A-4* mutant lines transformed with *RECQ4A-ΔN* growing on agar plates are shown. After germination and formation of cotyledons, the *top3A-1* mutant stops to grow. *top3A-2* mutant plants are smaller than wild type plants and show malformed, curled leafs. The growth of *recq4A-4* mutant lines expressing RECQ4A-ΔN is comparable with wild type and they did not show any growth defects. Scale bar = 1 cm.

Table S1. Cloning PCRs and primers used to create plasmids for *in vivo* studies. pPZP221 was used as a basis for the construction of the plant transformation vectors. The different *RECQ4A* constructs consist of a promoter region, a coding region and a terminator region. Amplification of the respective DNA fragment was performed by PCR using the depicted primer combinations and PCR templates. For the creation of homologous regions that are required for assembly of the single DNA fragments by In-Fusion cloning or overlap extension PCR, the DNA fragments were extended by PCR with the appropriate overhang primer. 4A *AtRECQ4A* sequences; 4B *AtRECQ4B* sequences; gDNA genomic DNA; cDNA copy DNA, ORF open reading frame; pro promoter; term terminator.

plasmid	*DNA fragment*	*template*	*PCR*	primer name	primer sequence
pPZP221-RECQ4A	*pro+*	*gDNA*	*amplification*	RQ4A-Pro-fw	5´-AAGATTCATGTATTTGATTTTTG-3´
				RQ4A-PrR	5´-GACCTCTAGGTTTCTGAAC-3´
	+pro+*	*pro+*	*extending with homologies*	RQ4A-Pro-fw-e-pPZP221BamHI	5´-CGGTACCCGGGGATCCAAGATTCATGTATTTGATTTTTG-3´
				RQ4A-PrR	5´-GACCTCTAGGTTTCTGAAC-3´
	+ORF+*	*cDNA*	*amplification*	RQ4A-ATG(+10)-fw	5´-GTCACCTCCCCGAAGTTC-3´
				RQL2-stop	5´-TCACCACGGTAATACTCTTC-3´
	+term	*gDNA*	*amplification*	RQ4A-Stop2-fw	5´-GAAGAGTATTACCGTGGTGA-3´
				RQ4A-Term-rev	5´-CAAAGCAAAACAAGCAGACAA-3´
	+term+*	*+term*	*extending with homologies*	RQ4A-Stop2-fw	5´-GAAGAGTATTACCGTGGTGA-3´
				RQ4A-Term-rev-e-pPZP221BamHI	5´-CGACTCTAGAGGATCCCAAAGCAAAACAAGCAGACAA-3´
pPZP221-RECQ4A-HD	*pro+*	*gDNA*	*amplification*	RQ4A-Pro-fw	5´-AAGATTCATGTATTTGATTTTTG-3´
				RQ4A-PrR	5´-GACCTCTAGGTTTCTGAAC-3´
	+ORF(HD)+	*cDNA*	*site directed mutagenesis,*	RQ4A-ATG-cDNA	5´-ATGAGTAGGAGTCACCTCC-3´
			OLE-PCR,	RQL2-stop	5´-TCACCACGGTAATACTCTTC-3´
			amplification	RQ4A-Mut-rev	5´-GTCAAACTCATCCCTCCTC-3´
				RQ4A-Mut-fw	5´-GAGGAGGGATGAGTTTGAC-3´
	+term	*gDNA*	*amplification*	RQ4A-Stop2-fw	5´-GAAGAGTATTACCGTGGTGA-3´
				RQ4A-Term-rev	5´-CAAAGCAAAACAAGCAGACAA-3´
	+ORF(HD)-term	*+ORF(HD)+ & +term*	*OLE-PCR*	RQ4A-ATG-cDNA	5´-ATGAGTAGGAGTCACCTCC-3´
				RQ4A-Term-rev	5´-CAAAGCAAAACAAGCAGACAA-3´
	pro-ORF(HD)-term ‡	*pro+ & +ORF(HD)-term*	*OLE-PCR*	RQ4A-Pro-fw	5´-AAGATTCATGTATTTGATTTTTG-3´
				RQ4A-Term-rev	5´-CAAAGCAAAACAAGCAGACAA-3´
pPZP221-RECQ4A-ΔN	*pro*	*gDNA*	*amplification*	RQ4A-Pro-fw	5´-AAGATTCATGTATTTGATTTTTG-3´
				RQ4A-Pro-Del-rev	5´-CATGGGTCTAACATGTTGAAG-3´
	+pro+*	*pro*	*extending with homologies*	RQ4A-Pro-fw-e-pPZP221BamHI	5´-CGGTACCCGGGGATCCAAGATTCATGTATTTGATTTTTG-3´
				ST-rev	5´-CAGGAGGCAGCATGGGTCTAACATGTTGAAG-3´
	ORF(ΔN)	*cDNA*	*amplification*	RQ4A-Del-fw	5´-CTGCCTCCTGAATTATGTTC-3´
				RQL2-stop	5´-TCACCACGGTAATACTCTTC-3´
	+ORF(ΔN)+*	*ORF(ΔN)*	*extending with homologies*	ST-fw	5´-TAGACCCATGCTGCCTCCTGAATTATGTTC-3´
				PJ-rev	5´-GGTCACAACTTCACCACGGTAATACTCTTC-3´
	term	*gDNA*	*amplification*	RQ4A-Term-fw	5´-AGTTGTGACCACTGAAATTTTAG-3´
				RQ4A-Term-rev	5´-CAAAGCAAAACAAGCAGACAA-3´
	+term+*	*term*	*extending with homologies*	PJ-fw	5´-ACCGTGGTGAAGTTGTGACCACTGAAATTTTAG-3´
				RQ4A-Term-rev-e-pPZP221BamHI	5´-CGACTCTAGAGGATCCCAAAGCAAAACAAGCAGACAA-3´
pPZP221-RECQ4A-HD-ΔN	*pro*	*gDNA*	*amplification*	RQ4A-Pro-fw	5´-AAGATTCATGTATTTGATTTTTG-3´
				RQ4A-Pro-Del-rev	5´-CATGGGTCTAACATGTTGAAG-3´
	+pro+*	*pro*	*extending with homologies*	RQ4A-Pro-fw-e-pPZP221BamHI	5´-CGGTACCCGGGGATCCAAGATTCATGTATTTGATTTTTG-3´
				ST-rev	5´-CAGGAGGCAGCATGGGTCTAACATGTTGAAG-3´
	ORF(HD-ΔN)-term	*pPZP221-K-RECQ4A-HD*	*amplification*	RQ4A-Del-fw	5´-CTGCCTCCTGAATTATGTTC-3´
				RQ4A-Term-rev	5´-CAAAGCAAAACAAGCAGACAA-3´
	+ORF(HD-ΔN)-term+*	*ORF(HD-ΔN)-term*	*extending with homologies*	ST-fw	5´-TAGACCCATGCTGCCTCCTGAATTATGTTC-3´
				RQ4A-Term-rev-e-pPZP221BamHI	5´-CGACTCTAGAGGATCCCAAAGCAAAACAAGCAGACAA-3´
pPZP221-RECQ-(4B)4A	*pro*	*gDNA*	*amplification*	RQ4A-Pro-fw	5´-AAGATTCATGTATTTGATTTTTG-3´
				RQ4A-Pro-rev vor ATG	5´-GGGTCTAACATGTTGAAGAG-3´
	+pro+*	*pro*	*extending with homologies*	RQ4A-Pro-fw-e-pPZP221BamHI	5´-CGGTACCCGGGGATCCAAGATTCATGTATTTGATTTTTG-3´
				4A-P/4B-Nt-rev	5´-TCACCACCATGGGTCTAACATGTTGAAGAG-3´
	5´-ORF(4B)	*cDNA*	*amplification*	RQL2A-(-1) ATG	5´-ATGGTGGTGACAAGAGGAG-3´
				RQ4B-Cr2	5´-CACTTTTTGTCATTTGAACTC-3´
	+5´-ORF(4B)+*	*5´-ORF(4B)*	*extending with homologies*	4A-P/4B-Nt-fw	5´-TGTTAGACCCATGGTGGTGACAAGAGGAG-3´
				OP-rev	5´-ACGACTGCTCCACTTTTTGTCATTTGAACTC-3´
	3´-ORF(4A)	*cDNA*	*amplification*	RQ4A-C2	5´-GAGCAGTCGTGAATTTCCC-3´
				RQL2-stop	5´-TCACCACGGTAATACTCTTC-3´
	+3´-ORF(4A)+*	*3´-ORF(4A)*	*extending with homologies*	OP-fw	5´-ACAAAAAGTGGAGCAGTCGTGAATTTCCC-3´
				PJ-rev	5´-GGTCACAACTTCACCACGGTAATACTCTTC-3´
	term	*gDNA*	*amplification*	RQ4A-Term-fw	5´-AGTTGTGACCACTGAAATTTTAG-3´
				RQ4A-Term-rev	5´-CAAAGCAAAACAAGCAGACAA-3´
	+term+*	*term*	*extending with homologies*	PJ-fw	5´-ACCGTGGTGAAGTTGTGACCACTGAAATTTTAG-3´
				RQ4A-Term-rev-e-pPZP221BamHI	5´-CGACTCTAGAGGATCCCAAAGCAAAACAAGCAGACAA-3´
pPZP221-RECQ-4A(4B)	*pro+*	*gDNA*	*amplification*	RQ4A-Pro-fw	5´-AAGATTCATGTATTTGATTTTTG-3´
				RQ4A-Pro-AS	5´-TTGATTCGAATTAATCATGGGT-3´
	+pro+*	*pro+*	*extending with homologies*	RQ4A-Pro-fw-e-pPZP221BamHI	5´-CGGTACCCGGGGATCCAAGATTCATGTATTTGATTTTTG-3´
				RQ4A-Pro-AS	5´-TTGATTCGAATTAATCATGGGT-3´
	+5´-ORF(4A)	*cDNA*	*amplification*	RQ4A-ATG-fw	5´-ATGATTAATTCGAATCAAATGAG-3´
				4A-Ct-rev	5´-AGAAAATAGTATGGTAGCCTT-3´
	+5´-ORF(4A)+*	*+5´-ORF(4A)*	*extending with homologies*	RQ4A-ATG-fw	5´-ATGATTAATTCGAATCAAATGAG-3´
				4A-Nl/4B-Ck-rev	5´-TCTGGCCTCCAGAAAATAGTATGGTAGCCTT-3´
	3´-ORF(4B)	*cDNA*	*amplification*	4B-Ct-fw	5´-GGAGGCCAGAGTATAACAAT-3´
				RQ4B-Stop2-rev	5´-TTATTTTTTGTTCTTCACTACC-3´
	+3´-ORF(4B)+*	*3´-ORF(4B)*	*extending with homologies*	4A-Nl/4B-Ck-fw	5´-ACTATTTTCTGGAGGCCAGAGTATAACAAT-3´
				IJ-rev	5´-GGTCACAACTTTATTTTTTGTTCTTCACTACC-3´
	term	*gDNA*	*amplification*	RQ4A-Term-fw	5´-AGTTGTGACCACTGAAATTTTAG-3´
				RQ4A-Term-rev	5´-CAAAGCAAAACAAGCAGACAA-3´
	+term+*	*term*	*extending with homologies*	IJ-fw	5´-CAAAAAATAAAGTTGTGACCACTGAAATTTTAG-3´
				RQ4A-Term-rev-e-pPZP221BamHI	5´-CGACTCTAGAGGATCCCAAAGCAAAACAAGCAGACAA-3´

\+ *indicates existing homologies to the adjacent fragment of the construct or to the linearized plasmid backbone*

* *indicates starting material for a single In-Fusion reaction with pPZP221 linearized with BamHI to create the respective pPZP221 derivative*

‡ *indicates starting material for cloning into pCR-Blunt II-TOPO (Invitrogen), followed by a subcloning into pPZP221 using BamHI and PstI restriction sites surrounding the construct*
